# Supplementary material for: Light and dark conditions control the nitrous oxide uptake and emission dynamics in a subarctic, nutrient-poor permafrost peatland
Source: Commun Earth Environ. 2026 May 30;7(1):471. doi: 10.1038/s43247-026-03698-3 (PMC13222127; doi:10.1038/s43247-026-03698-3)
Supplement: Supplementary file 2 — Supplementary Information [file 43247_2026_3698_MOESM2_ESM.pdf]

Light and dark conditions control the nitrous oxide uptake and emission dynamics in a subarctic, nutrient-poor permafrost peatland

Nathalie Ylenia Triches[*1,2], Abdullah Bolek[1], Mirkka Rovamo[3,4], Richard E. Lamprecht[3], Kseniia Ivanova[1], Wasi Hashmi[3], Theresia Yazbeck[1], Nicholas James Eves[1], Dhiraj Paul[3], Anna-Maria Virkkala[5,6], Timo Vesala[2,7], Christina Biasi[3,8], Maija E. Marushchak[3], Mathias Göckede[1]

[1]Max Planck Institute for Biogeochemistry, Jena, Germany
[2]Institute for Atmospheric and Earth System Research/Forest Sciences, Faculty of Agriculture and Forestry, University of Helsinki, Helsinki, Finland
[3]Department of Environmental and Biological Sciences, Faculty of Science, Forestry and Technology, University of Eastern Finland, Kuopio, Finland
[4]Business School, Faculty of Social Sciences and Business Studies, University of Eastern Finland, Kuopio, Finland
[5]Woodwell Climate Research Center, Falmouth, USA
[6]Finnish Meteorological Institute, Helsinki, Finland
[7]Institute for Atmospheric and Earth System Research/Physics, Faculty of Science, University of Helsinki, Helsinki, Finland
[8]University of Innsbruck, Institute of Ecology, Innsbruck, Austria

Supplementary Information

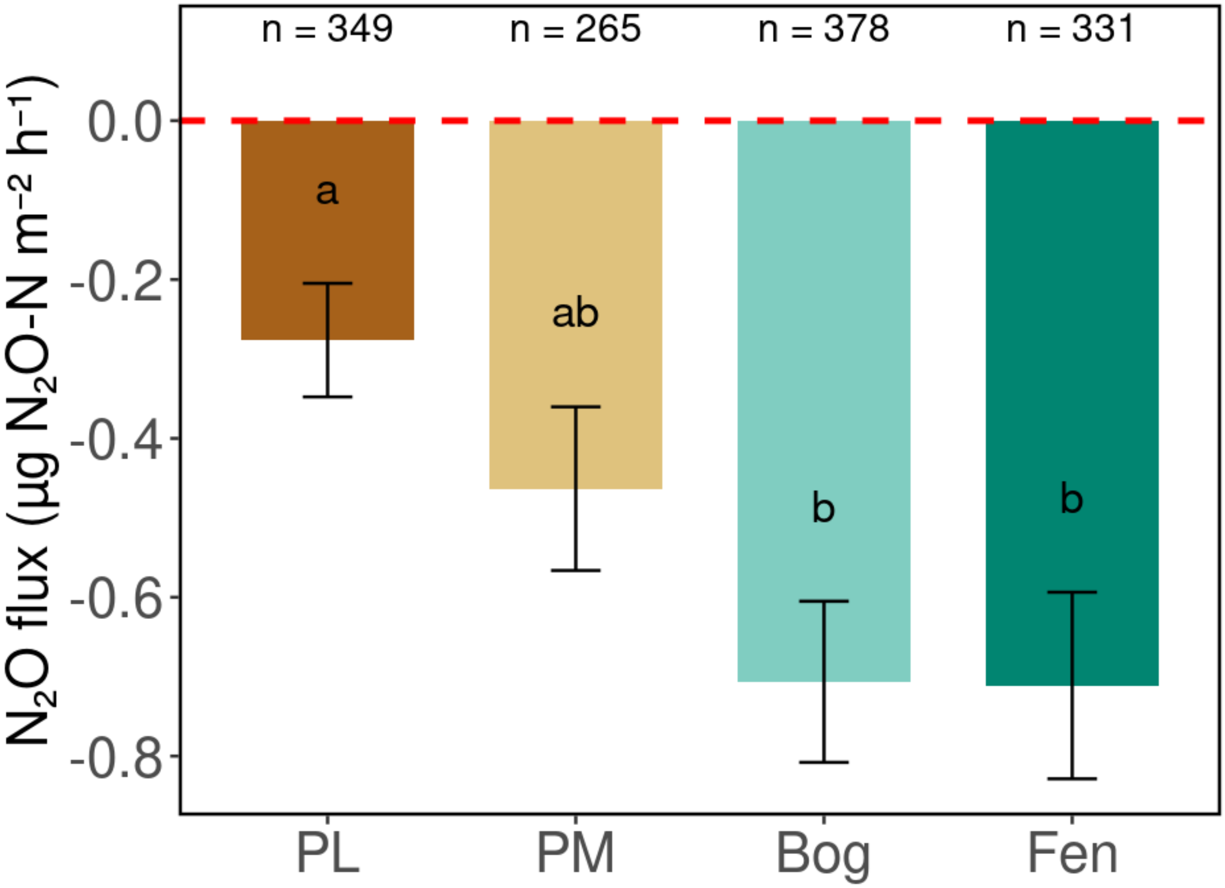

Figure S1: N_2O flux over all measurement campaigns and micro habitats, excluding one hot spot, with PL, PM indicating Palsa lichen and Palsa moss, respectively. Letters indicate significance according to ANOVA and Tukey HSD post-hoc tests, with differing letters between micro habitats indicating significant differences. The dashed red horizontal line indicates the border between a source (positive values) and sink (negative values).

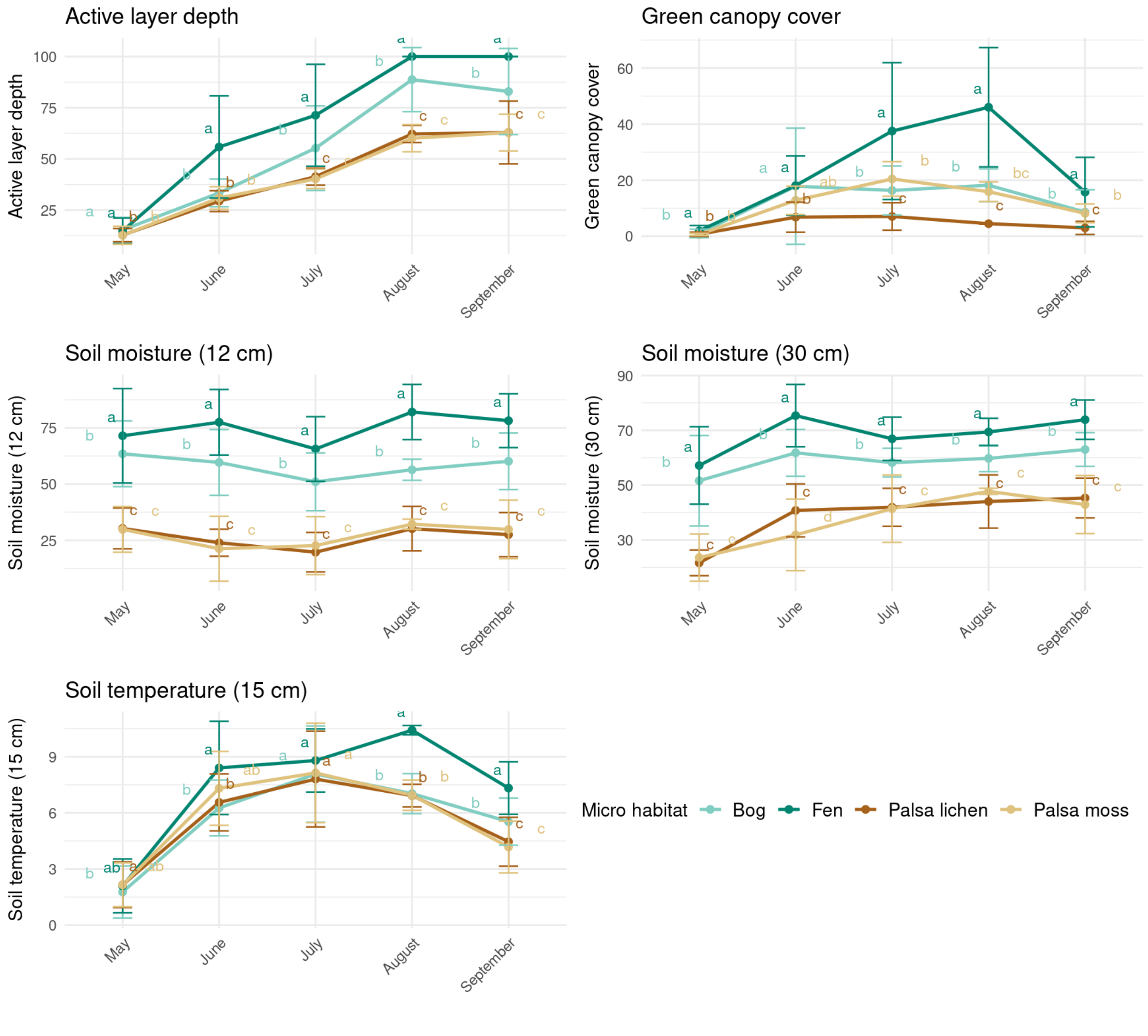

Group letters (a, b, c...) indicate statistically significant differences (Tukey HSD, $p < 0.05$).

Figure S2: Monthly means (error bars indicating $\pm$ standard deviation) of active layer depth, green canopy cover, soil moisture at 12 cm and 30 cm, and soil temperature at 15 cm between May and September, divided into palsa lichen, palsa moss, bog, and fen habitats. Group letters (a,b,c,...) indicate statistically significant differences to other micro habitats (ANOVA and Tukey HSD post-hoc test, $p < 0.05$).

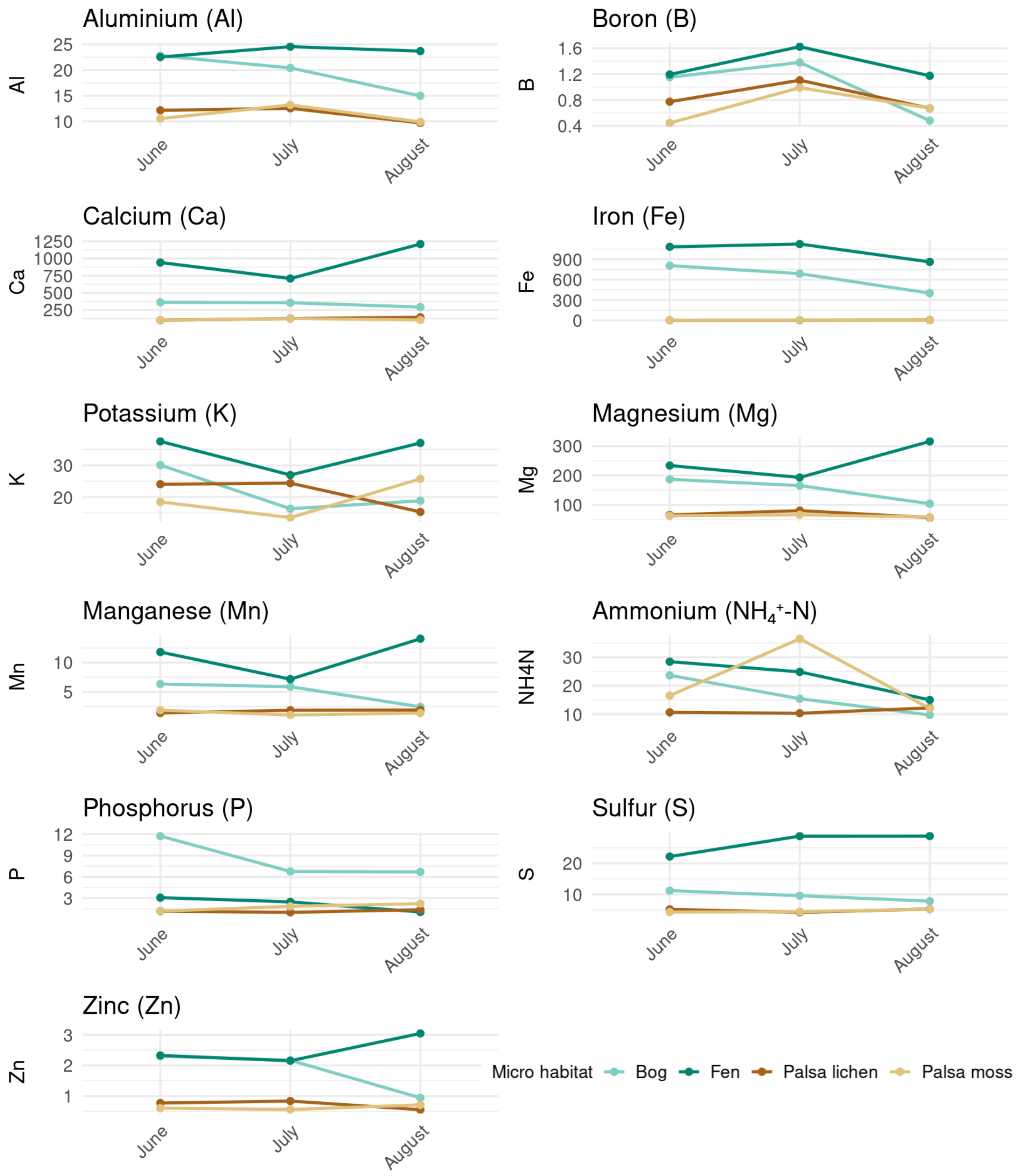

Figure S3: Nutrient values of aluminium (Al), boron (B), calcium (Ca), iron (Fe), potassium (K), magnesium (Mg), Manganese (Mn), Ammonium (NH_4^+), phosphorus (P), sulfur (S) and zinc (Zn) per micro habitat in μg nutrient / 10 cm^3 / burial time (3 weeks).

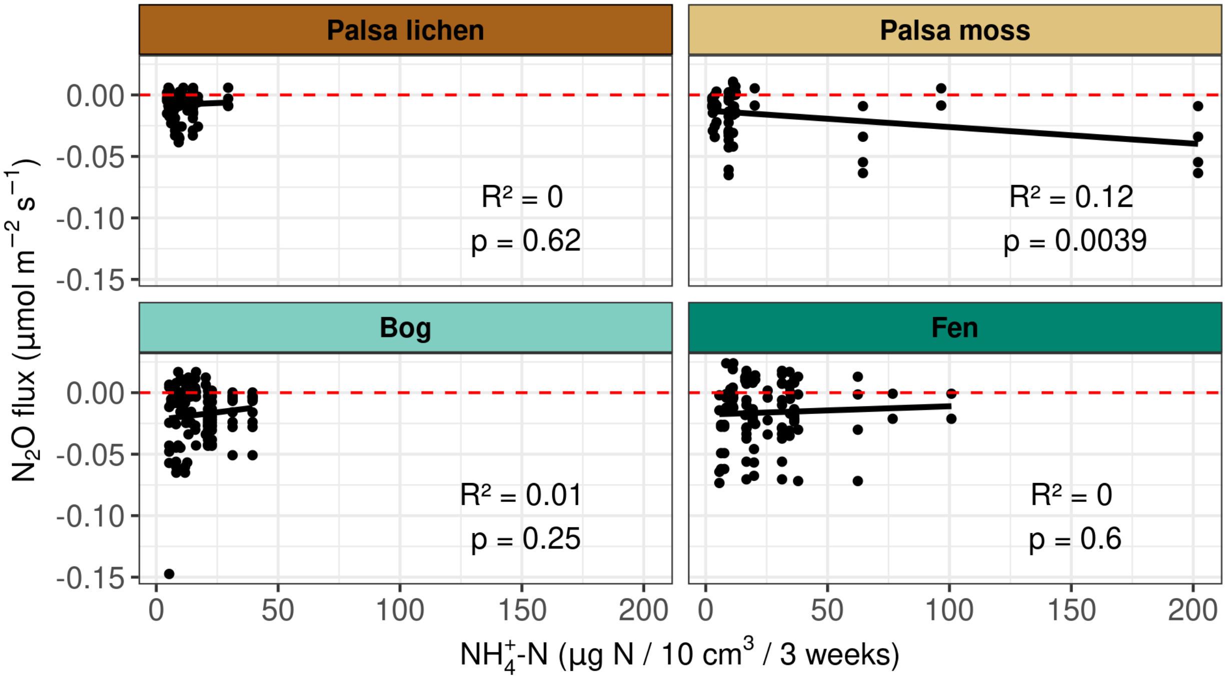

Figure S4: Regression analysis between measured NH_4^+ and N_2O fluxes divided into the different micro habitats (excluding the continuous hot spot), with R^2 and p-values.

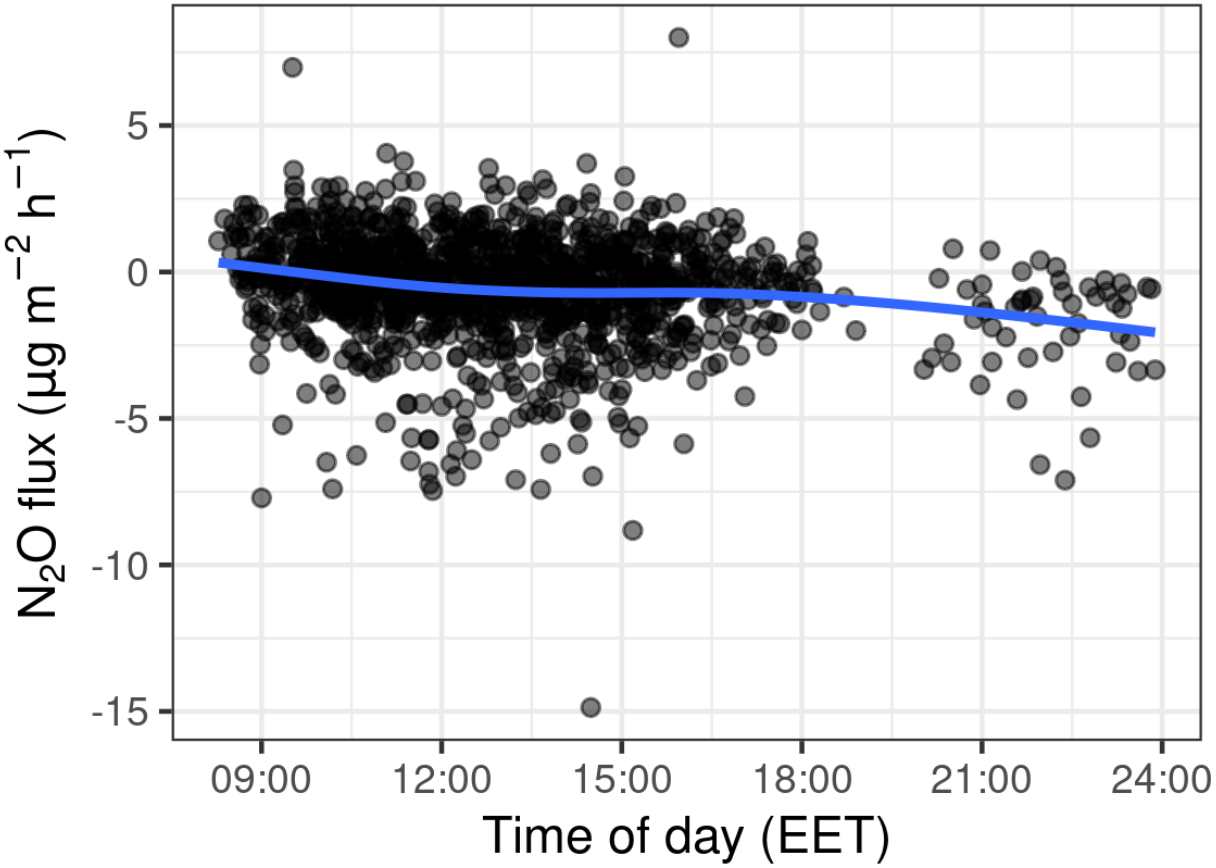

Figure S5: Diurnal cycle of N_2O fluxes from all micro habitats (excluding hot spot) during all measurement campaigns in time of day (EET). 4 measurements shortly after 24:00 were removed for improved visualisation.

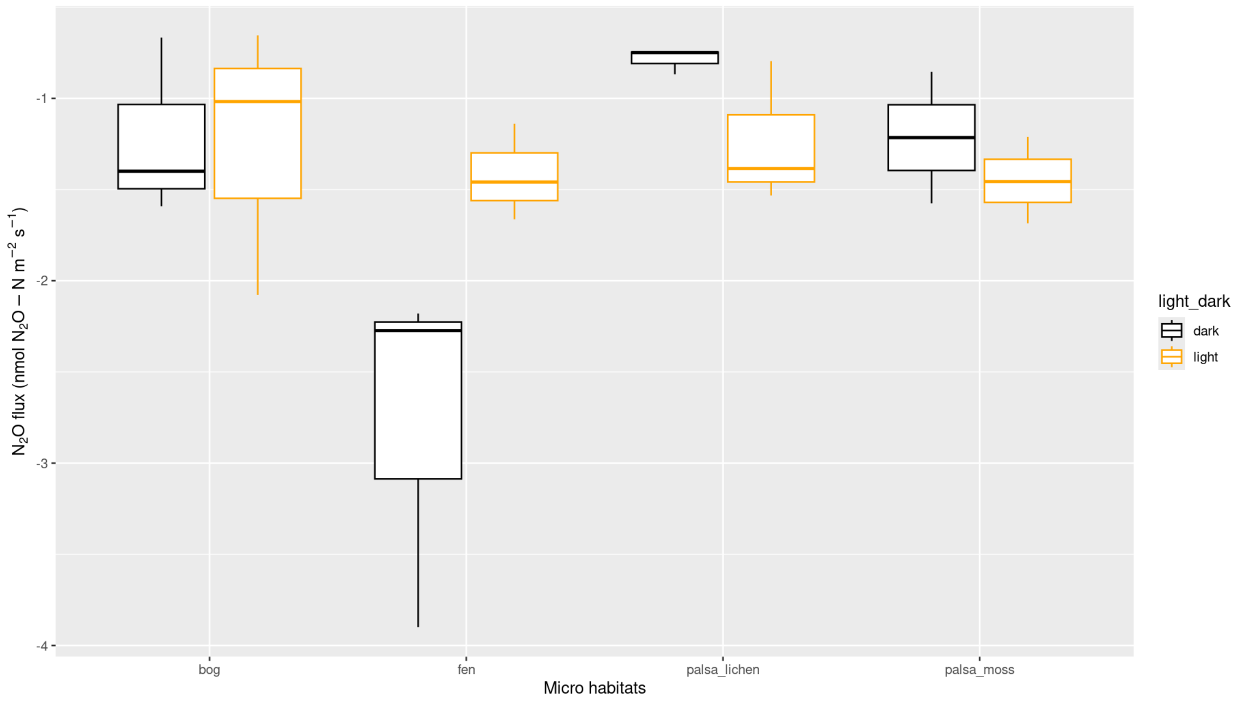

Figure S6: Comparison between light (organge) and dark (grey) mobile chamber measurements in the nearby palsa mire Storflakket.

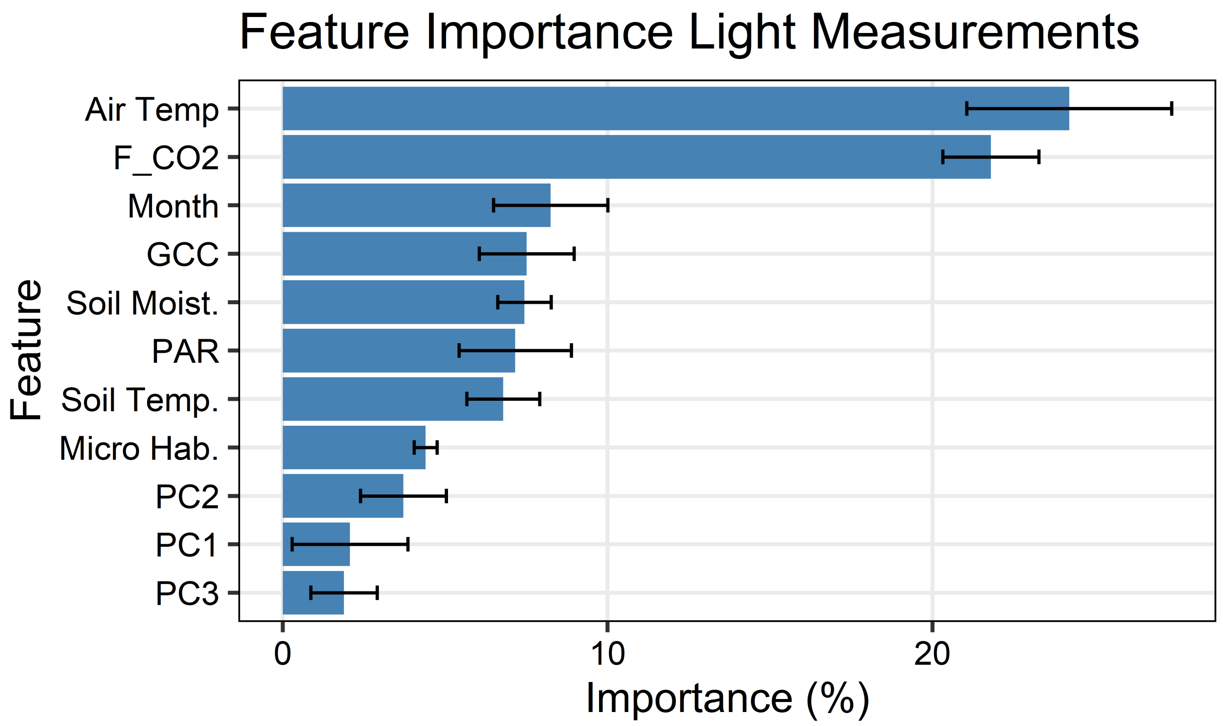

Figure S7: Importance of environmental variables ("features") **and nutrients during light measurements**, with F_CO2 = NEE, Air Temp. = air temperature, Month = measurement campaign month, Soil Moist. = soil moisture, PAR = photosynthetically active radiation, GCC = green canopy cover, Micro Hab. = micro habitat, and Soil Temp. = soil temperature

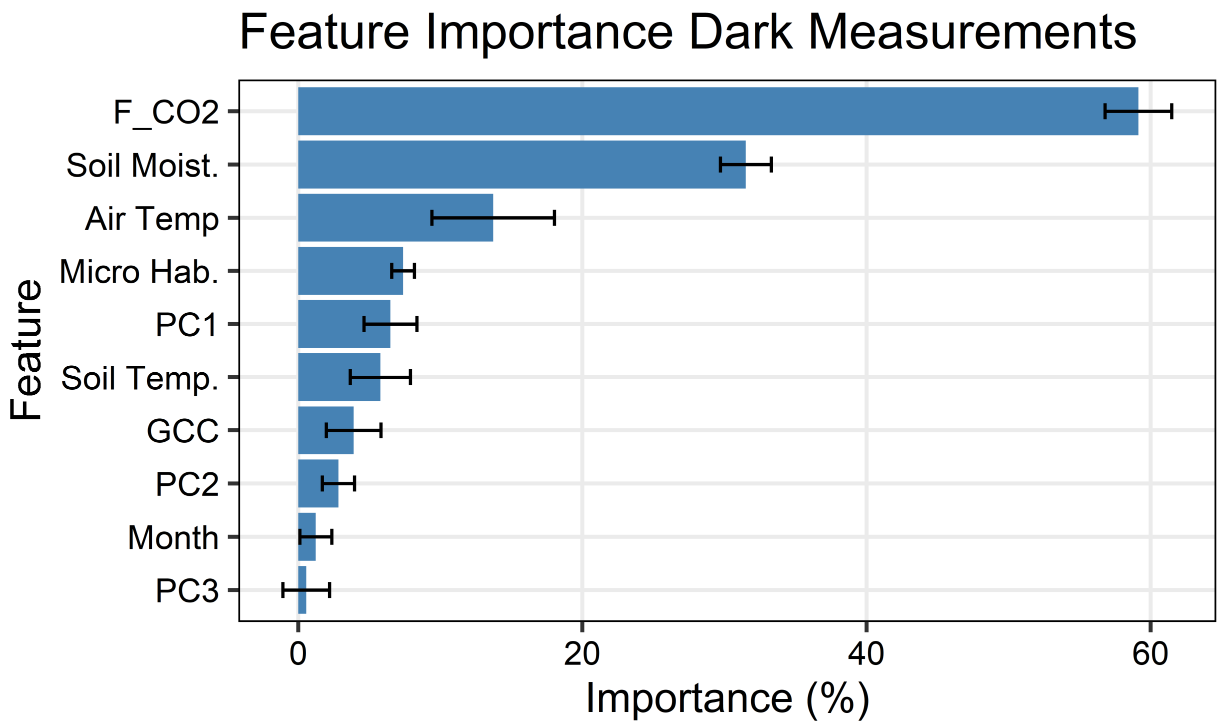

Figure S8: Importance of environmental variables (”features”) **and nutrients during dark measurements**, with F_CO2 = NEE, Air Temp. = air temperature, Month = measurement campaign month, Soil Moist. = soil moisture, PAR = photosynthetically active radiation, GCC = green canopy cover, Micro Hab. = micro habitat, and Soil Temp. = soil temperature

Table S1: Number of replicates per plot_no, micro habitat, and exact coordinates of the final 24 chamber base position

Transect	Plot	Micro habitat	n replicates	Y coordinate	X coordinate
1	2	Palsa lichen	58	68.35588	19.04357
1	7	Bog	59	68.35595	19.04360
1	8	Palsa moss	79	68.35601	19.04387
1	10	Fen	62	68.35601	19.04411
1	11	Palsa lichen	72	68.35604	19.04403
1	12	Bog	72	68.35606	19.04402
2	15	Palsa moss	58	68.35597	19.04599
2	17	Palsa moss	62	68.35608	19.04625
2	18	Palsa lichen	62	68.35610	19.04619
2	19	Fen	76	68.35607	19.04681
2	20	Bog	76	68.35612	19.04652
2	23	Fen	57	68.35619	19.04701
3	25	Palsa lichen	52	68.35770	19.05122
3	26	Bog	48	68.35771	19.05117
3	27	Palsa moss	48	68.35771	19.05109
3	28	Bog	56	68.35760	19.05101
3	29	Palsa lichen	48	68.35763	19.05102
3	30	Palsa moss	41	68.35761	19.05116
3	31	Bog	55	68.35763	19.05152
3	32	Palsa moss	46	68.35766	19.05167
3	33	Palsa lichen	46	68.35767	19.05174
3	34	Fen	49	68.35793	19.05116
3	35	Fen	38	68.35801	19.05128
3	36	Fen	34	68.35806	19.05139
